# Supplementary material for: Design of a two-dimensional interplanar heterojunction for catalytic cancer therapy
Source: Nat Commun. 2022 May 3;13:2425. doi: 10.1038/s41467-022-30166-1 (PMC9065124; doi:10.1038/s41467-022-30166-1)
Supplement: Supplementary file 1 — Supplementary Information [file 41467_2022_30166_MOESM1_ESM.pdf]

Design of A Two-Dimensional Interplanar Heterojunction For Catalytic Cancer Therapy

Yong Kang[1†], Zhuo Mao[1†], Ying Wang[2], Chao Pan[1], Meitong Ou[2], Hanjie Zhang[3], Weiwei Zeng[3], Xiaoyuan Ji[1,*]

[1]Academy of Medical Engineering and Translational Medicine, Medical College, Tianjin University, Tianjin 300072, China.

[2]School of Pharmaceutical Sciences (Shenzhen), Sun Yat-sen University, Guangzhou 510275, China

[3]Tianjin Key Laboratory of Biomedical Materials, Key Laboratory of Biomaterials and Nanotechnology for Cancer Immunotherapy, Institute of Biomedical Engineering, Chinese Academy of Medical Sciences and Peking Union Medical College, Tianjin 300192, China.

[†]These authors contributed equally to this work.

[*]Corresponding author.

Email: jixiaoyuan@tju.edu.cn (X. Ji)

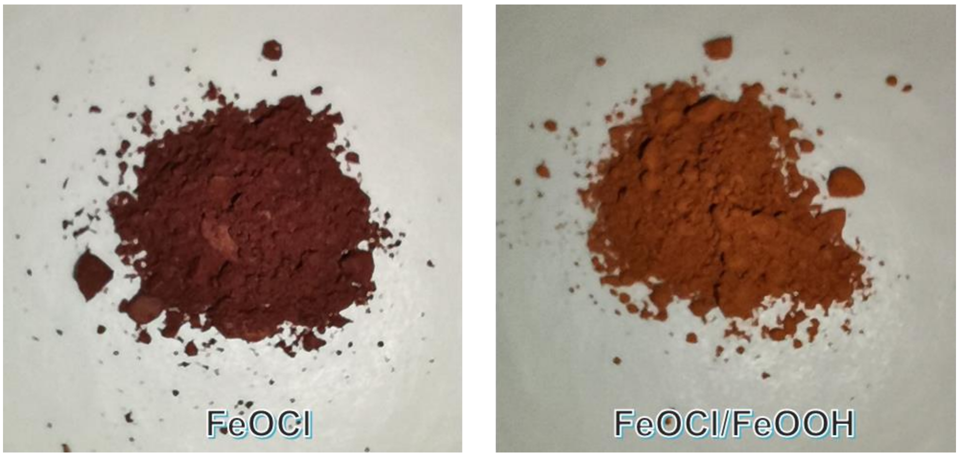

Supplementary Figure 1. Photo images of FeOCl and FeOCl/FeOOH powders.

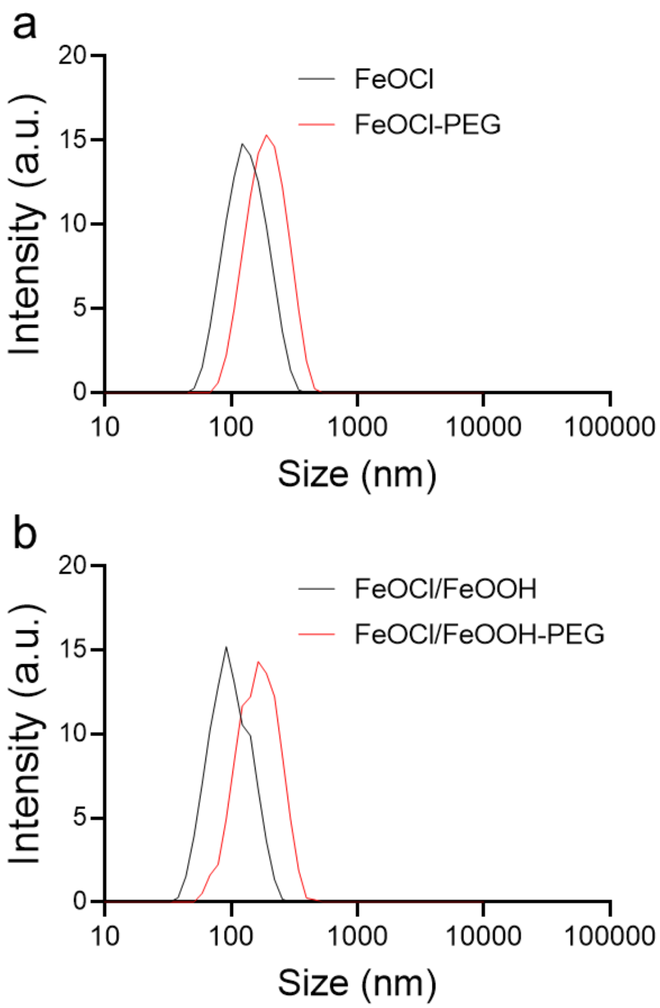

Supplementary Figure 2. Size distribution of a) FeOCl NSs and PEG modified FeOCl NSs and b) FeOCl/FeOOH NSs and PEG modified FeOCl/FeOOH NSs. Three times each experiment was repeated independently with similar results.

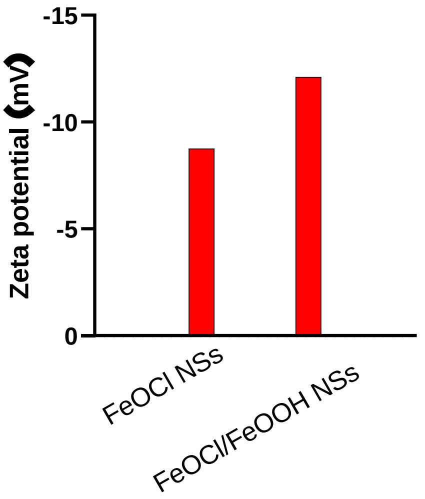

Supplementary Figure 3. The Zeta potentials of prepared FeOCl NSs and FeOCl/FeOOH NSs. Three times each experiment was repeated independently with similar results.

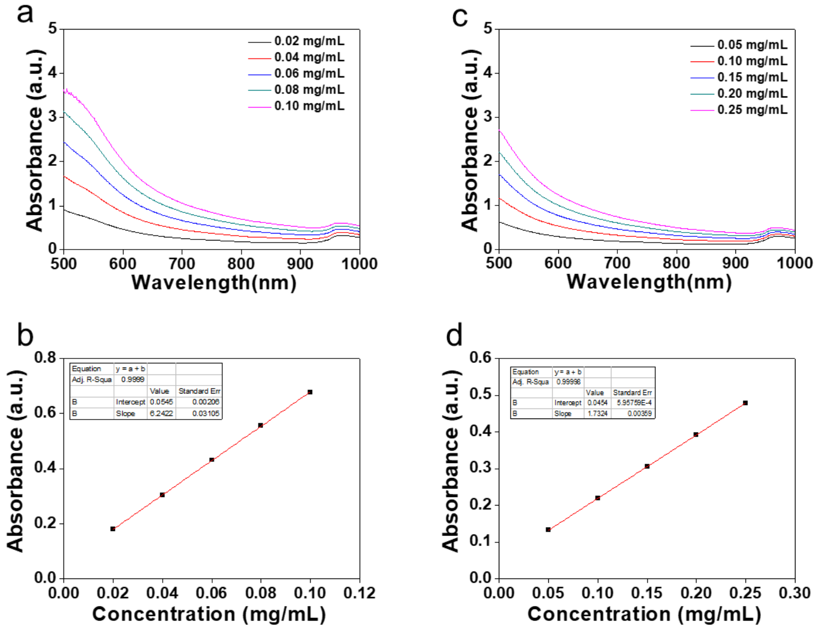

Supplementary Figure 4. UV-vis-NIR absorbance spectra of a) PEGylation FeOCl NSs and b) PEGylation FeOCl/FeOOH NSs dispersed in water at different concentrations. Three times each experiment was repeated independently with similar results. Normalized absorbance intensity of c) PEGylation FeOCl NSs and d) PEGylation FeOCl/FeOOH NSs divided by the characteristic length of the cell (A/L) at different concentrations for λ=808 nm.

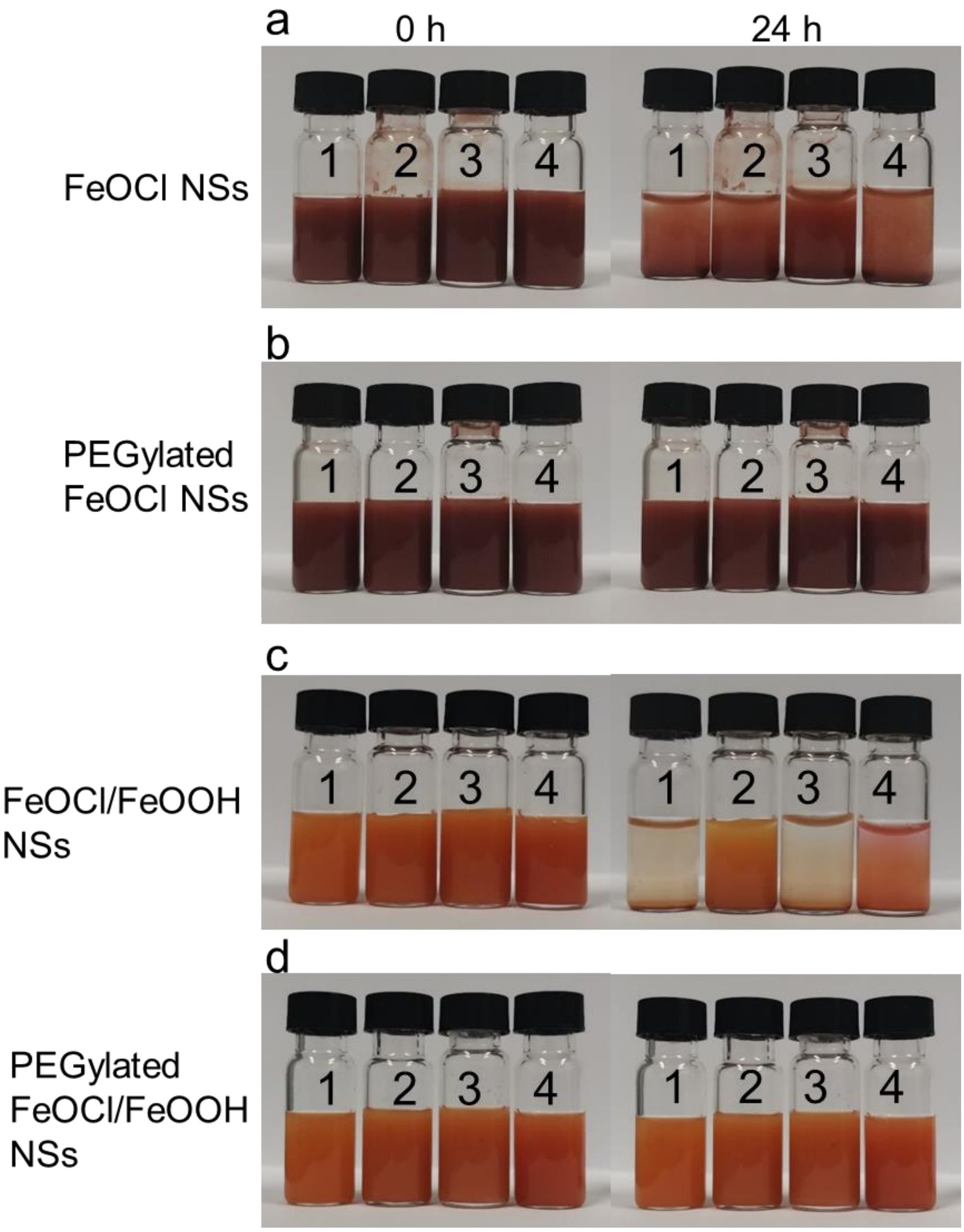

Supplementary Figure 5. The distribution of **a** FeOCl NSs, **b** PEGylated FeOCl NSs, **c** FeOCl/FeOOH NSs, and **d** PEGylated FeOCl/FeOOH NSs in (1) water, (2) PBS, (3) fetal bovine serum solution, and (4) medium.

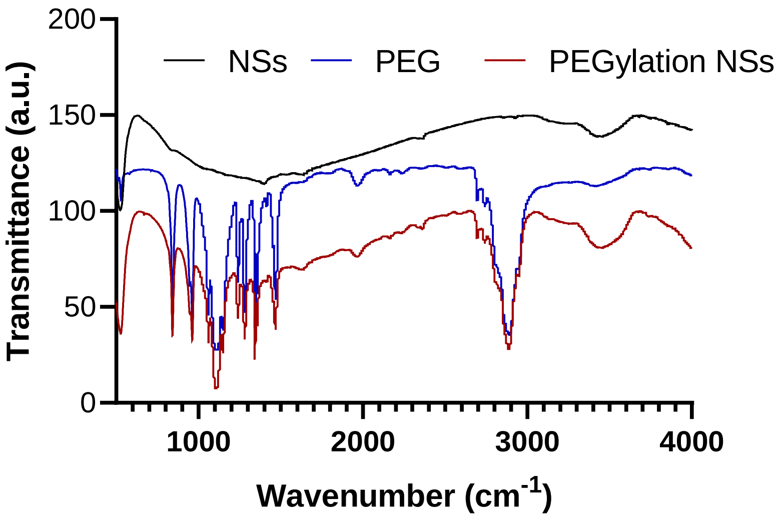

Supplementary Figure 6. FTIR spectra of PEG-NH_2, FeOCl/FeOOH NSs, and PEG modified FeOCl/FeOOH NSs. Three times each experiment was repeated independently with similar results.

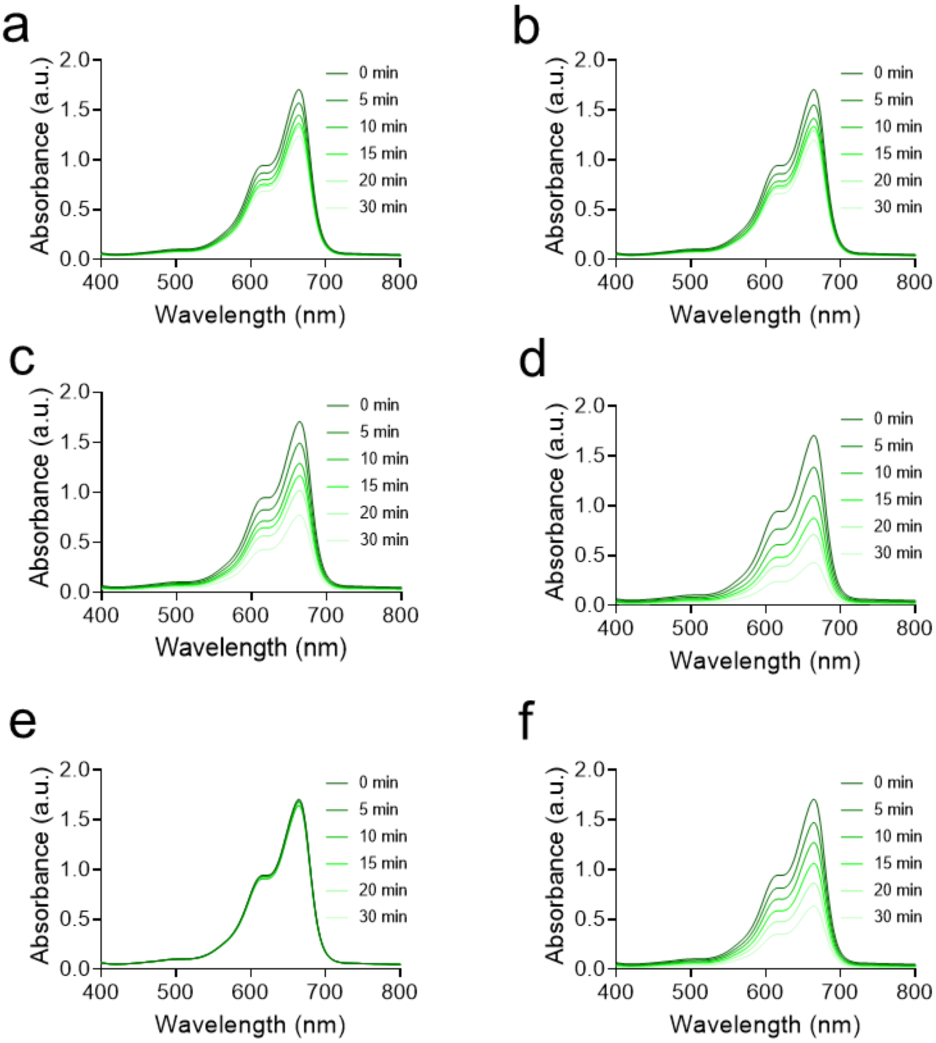

Supplementary Figure 7. Degradation of MB at different time points of treatment with FeOCl NSs or FeOCl/FeOOH NSs and different treatments: a) FeOCl NSs, b) FeOCl/FeOOH NSs, c) FeOCl NSs+US, d) FeOCl/FeOOH NSs+US, e) FeOCl NSs+US without H_2O_2, f) FeOCl/FeOOH NSs+US without H_2O_2. Three times each experiment was repeated independently with similar results.

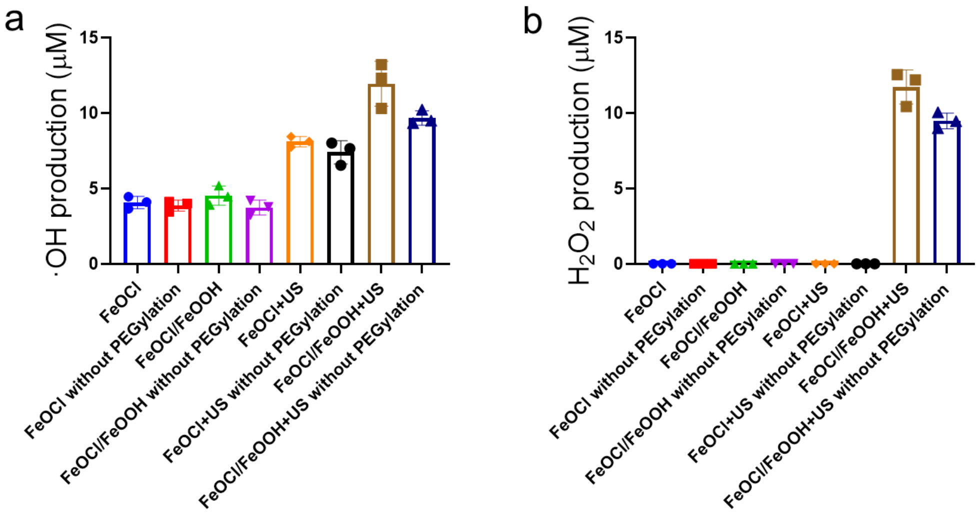

Supplementary Figure 8. The **a** ·OH and **b** H_2O_2 production data catalyzed by FeOCl NSs and FeOCl/FeOOH NSs with or without US irradiation and with or without PEGylation. Data are presented as mean ± s.d. (n = 3 independent experiments). Source data are provided as a Source Data file.

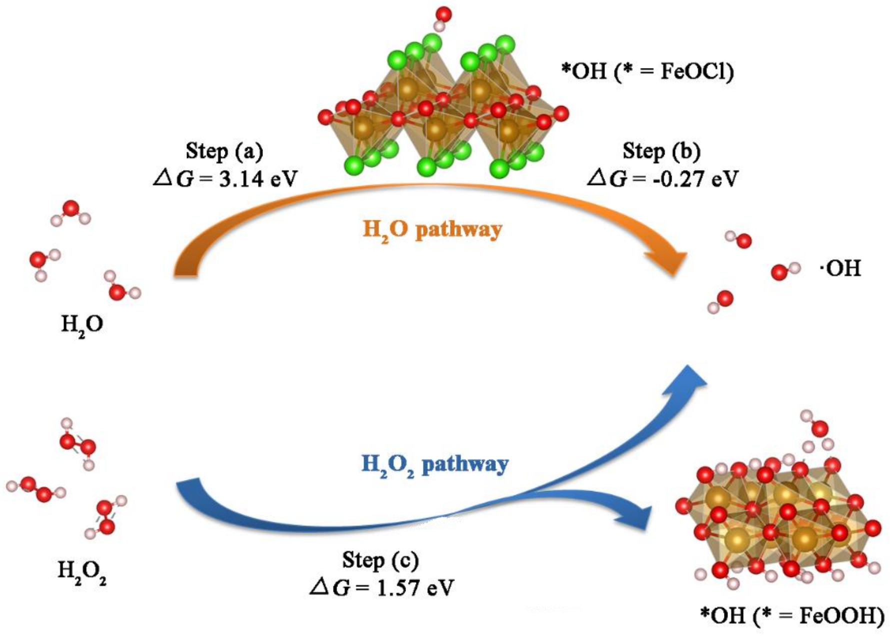

Supplementary Figure 9. Schematic diagram of catalytic pathways for ·OH generation. The free energy difference of each elementary reaction is shown, and the energy of the initial component is set to be 0 eV.

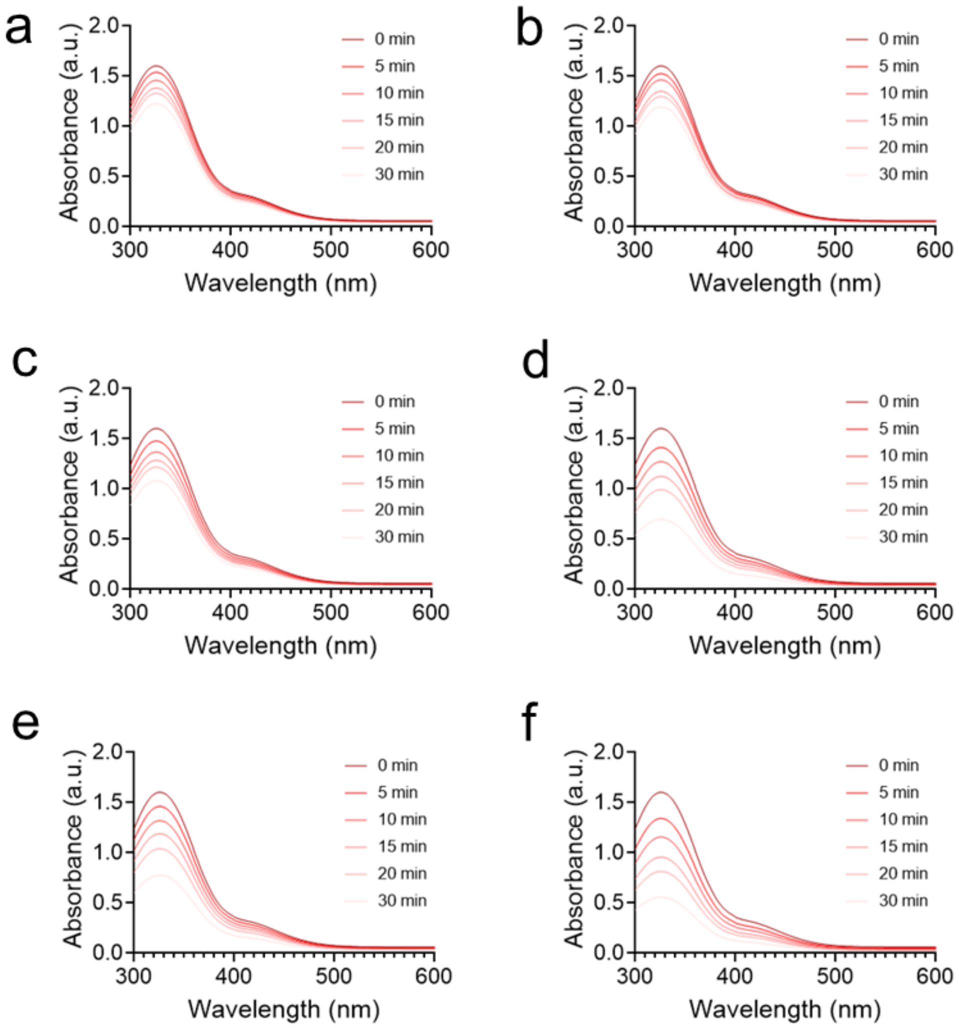

Supplementary Figure 10. Oxidization of GSH at different time points of treatment with FeOCl NSs or FeOCl/FeOOH NSs and different treatments: a) FeOCl NSs, b) FeOCl/FeOOH NSs, c) FeOCl NSs+US, d) FeOCl/FeOOH NSs+US, e) FeOCl NSs+US without H_2O_2, f) FeOCl/FeOOH NSs+US without H_2O_2. Three times each experiment was repeated independently with similar results.

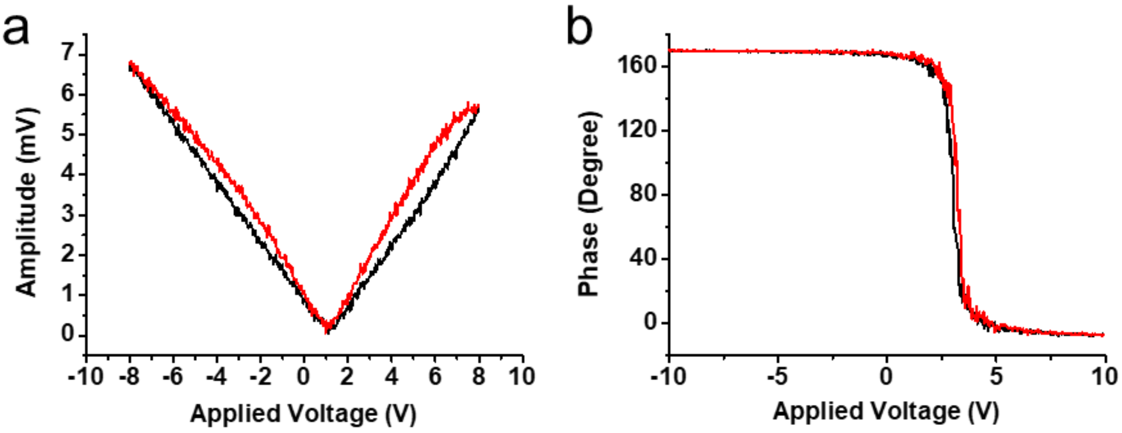

Supplementary Figure 11. **a** PFM amplitude butterfly loop and **b** PFM phase hysteresis loop of the FeOCl/FeOOH NSs. Three times each experiment was repeated independently with similar results.

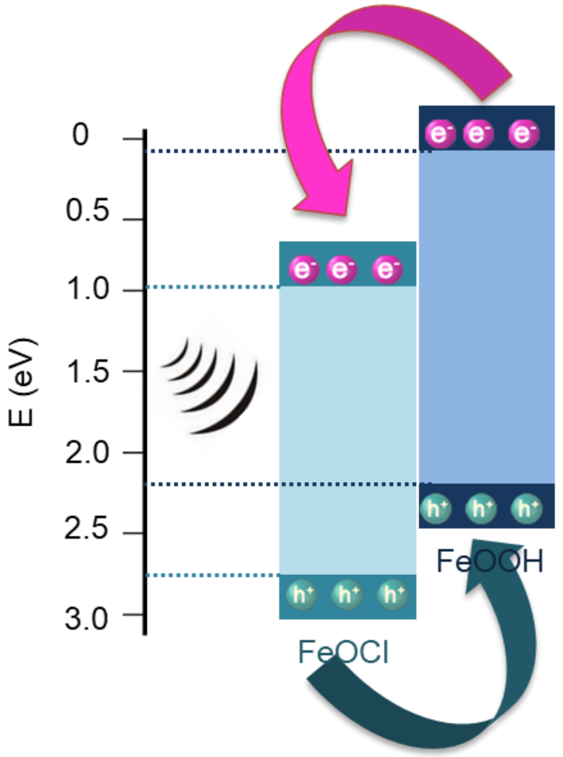

Supplementary Figure 12. Type II charges transfer in this FeOCl/FeOOH based heterojunction.

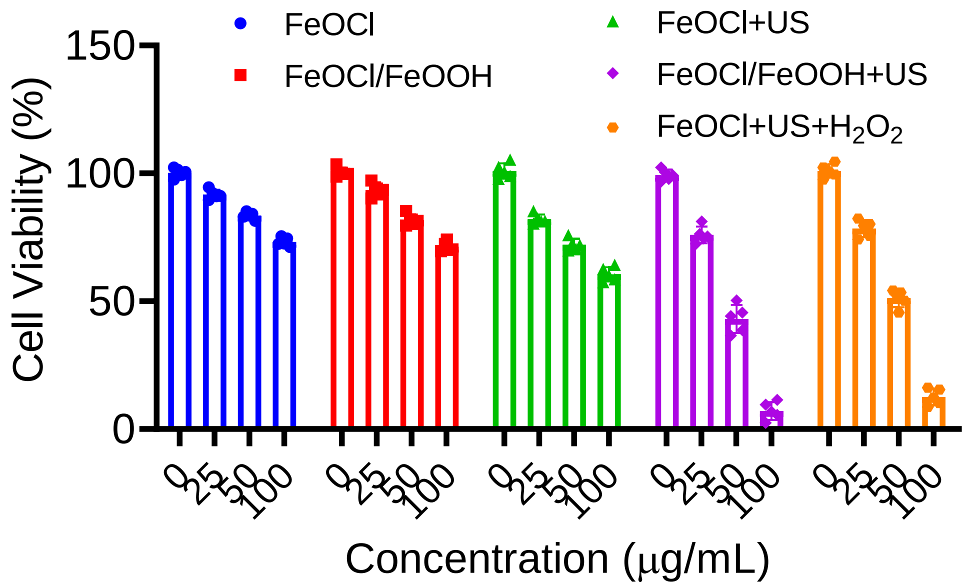

Supplementary Figure 13. Antitumor effect of FeOCl NSs or FeOCl/FeOOH NSs based fenton-like reaction under different treatments. Data are presented as mean ± s.d. (n = 5 biologically independent cells). Source data are provided as a Source Data file.

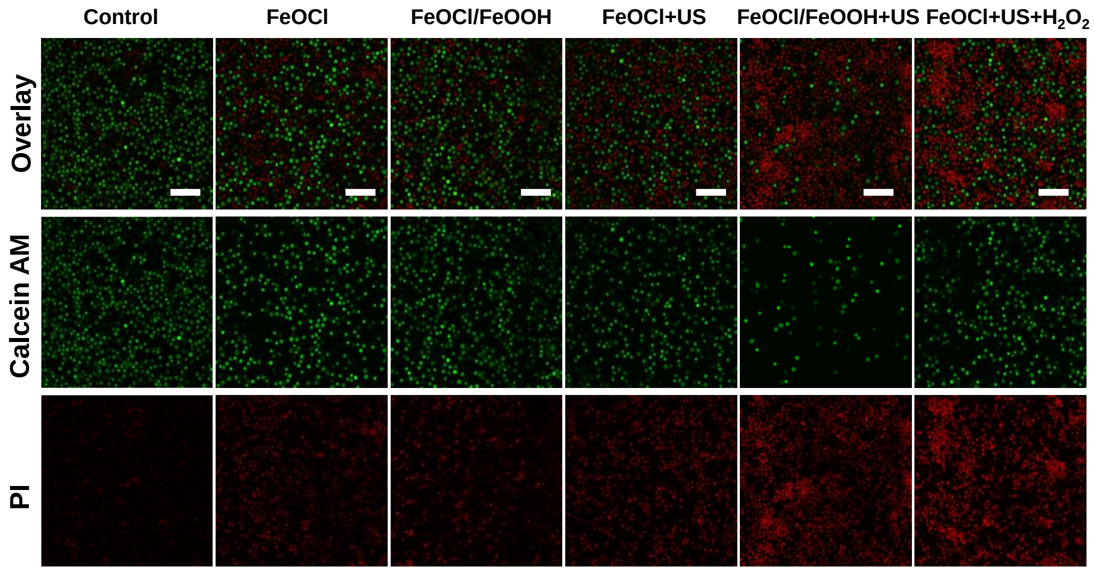

Supplementary Figure 14. Fluorescence images of MCF7 cells stained with Calcein-AM (live cells, green fluorescence) and PI (dead cells, red fluorescence) after treated with different conditions (scale bar = 150 μm). For these fluorescence images of MCF7 cells after different treatments, three times each experiment was repeated independently with similar results.

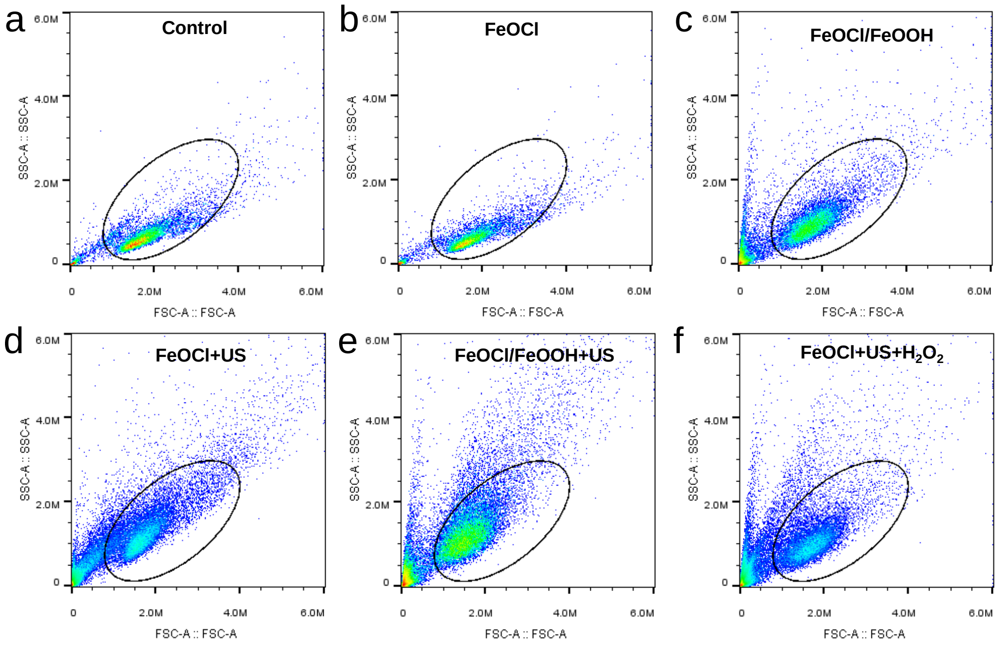

Supplementary Figure 15. FCM images of MCF7 cells after incubation with FeOCl NSs and FeOCl/FeOOH NSs under different treatments for 12 h. The cells gated in the images were distinguished as target cell group to be further analyzed. These gating panels correspond to Figure 6f in the manuscript.

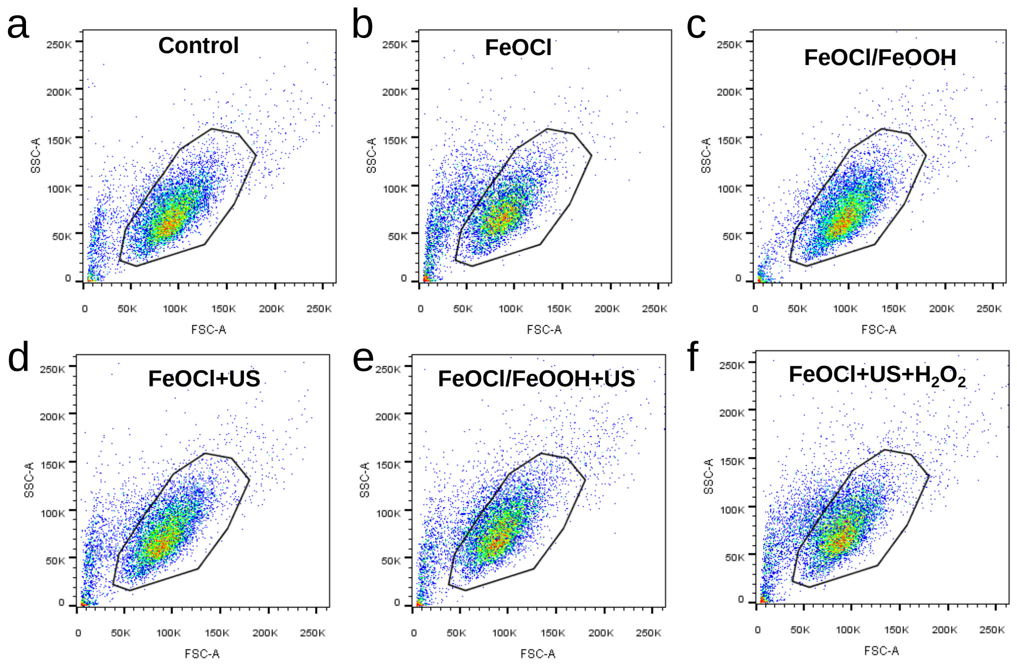

Supplementary Figure 16. FCM images of intracellular ROS generation. The cells gated in the images were distinguished as target cell group to be further analyzed. These gating panels correspond to Figure 7e in the manuscript.

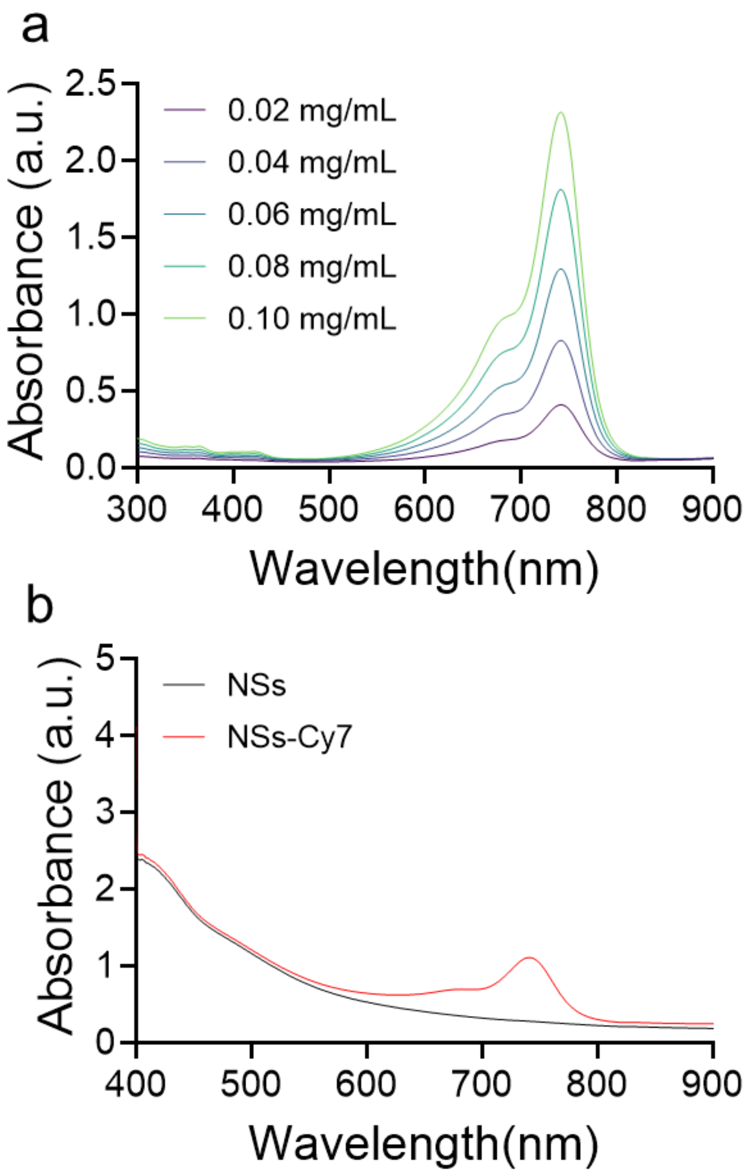

Supplementary Figure 17. a) UV-vis-NIR absorbance spectra of Cy 7 dispersed in water at different concentrations. b) UV-vis-NIR absorbance spectra of FeOCl/FeOOH NSs and Cy 7 loaded FeOCl/FeOOH NSs. Three times each experiment was repeated independently with similar results.

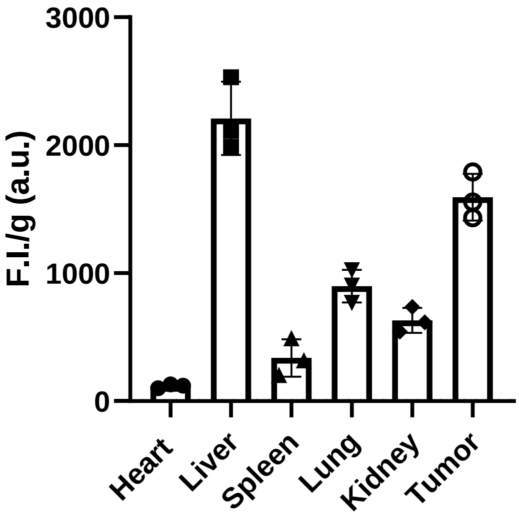

Supplementary Figure 18. Semiquantitative biodistribution of FeOCl/FeOOH NSs in tumor and major organs 24 h post-injection. Data are presented as mean ± s.d. (n = 3 biologically independent mice). Source data are provided as a Source Data file.

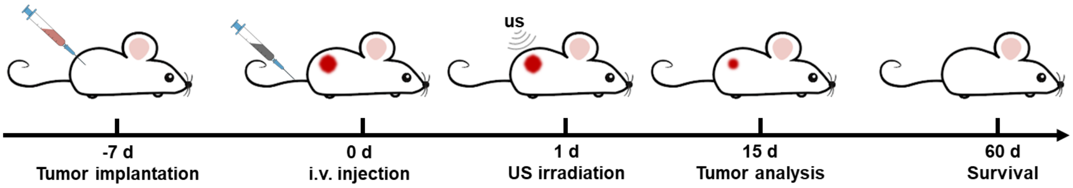

Supplementary Figure 19. Schematic diagram of treatment based on FeOCl/FeOOH NSs.

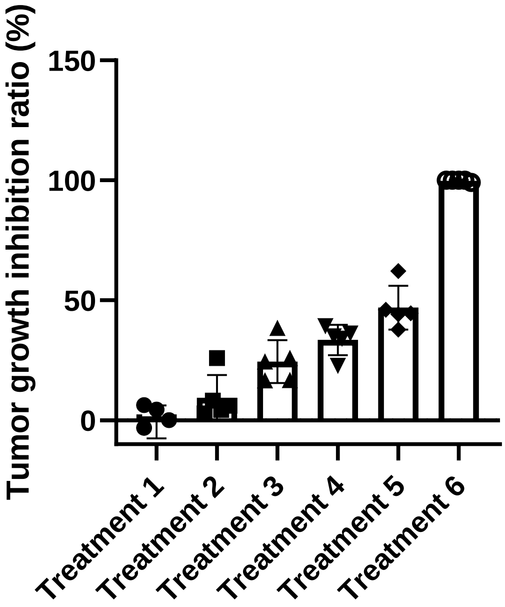

Supplementary Figure 20. Tumor growth inhibition ratio of each group. Data are presented as mean ± s.d. (n = 5 biologically independent mice). Source data are provided as a Source Data file.

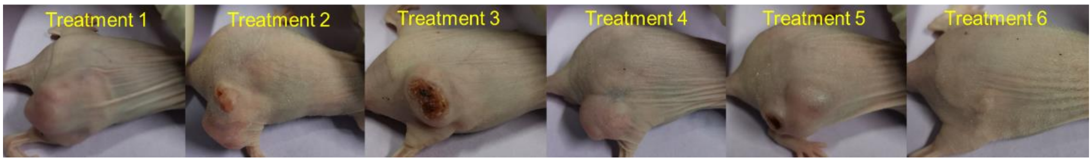

Supplementary Figure 21. Representative tumor photos in different groups after 14 days of treatment. For these tumor photos of different groups, five times each experiment was repeated independently with similar results. These representative tumor photos correspond to Figure 8e in the manuscript.

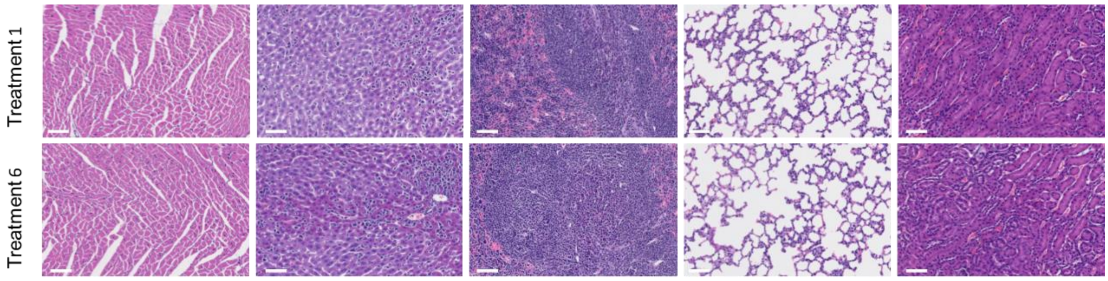

Supplementary Figure 22. HE images of major organs (heart, liver, spleen, lung, and kidney) after different treatments. For these HE images of major organs of different groups, five times each experiment was repeated independently with similar results.

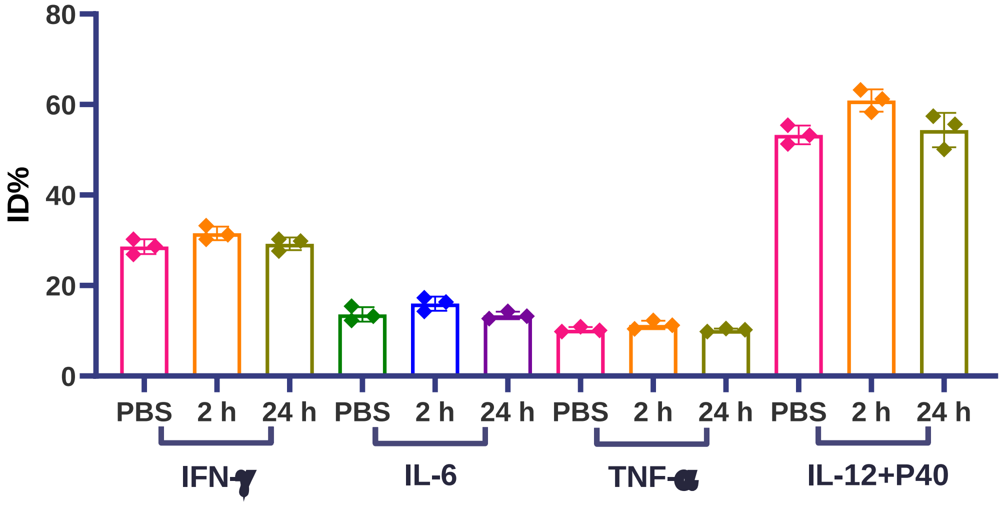

Supplementary Figure 23. Serum levels of IL-6, IFN-γ, TNF-α, and IL-12+P40 in mice at 2 and 24 h post i.v. injection of PBS versus FeOCl/FeOOH NSs. Data are presented as mean ± s.d. (n = 3 biologically independent mice).

Supplementary Table 1. Equilibrium distance (*d*) between nanosheet and H_2O. The bond angle (θ) and bond length (l_1, l_2) of H_2O, respectively. The adsorption energy (E_{ad}) and charge transfer (*Q*) of each adsorption systems.

**System**	***d*** **(Å)**	l_1 **(Å)**	l_2 **(Å)**	θ **(°)**	E_{ad} **(eV)**	***Q*** **(e)**
H_2O (isolated)	-	0.972	0.972	104.662	-	-
FeOCl-H_2O	0.50	0.972	0.973	102.883	-0.13	0.001
FeOOH-H_2O	1.90	0.974	1.101	105.366	-0.83	-0.006

Supplementary Table 2. Adsorption energy between nanosheet and H_2O.

**System**	$E_{complex}$ **(eV)**	$E_{substrate}$ **(eV)**	E_{H2O} **(eV)**	E_{ad} **(eV)**
FeOCl-H_2O	0.50	0.972	0.973	102.883
FeOOH-H_2O	1.90	0.974	1.101	105.366

Supplementary Table 3. Charge transfer between nanosheet and H_2O.

**System**	H_2O_adsorbed (e)	H_2O_isolated (e)	△Q
FeOCl-H_2O	8.0013	8	0.0013
FeOOH-H_2O	7.9938	8	-0.0062

Supplementary Table 4. Gibbs free energy calculation details.

**Step (a)**						
	*OH	H_2	*	H_2O		
**E**	-145.20845	-6.77083	-137.33142	-14.22331	△E	2.960865
**E_{ZPE}**	0.25561	0.26875	0	0.5677	△E_{ZPE}	-0.177715
**S**	0.00015	0.00136	0	0.00197	△S	-0.00114
					△G	3.136721
**Step (b)**						
	*OH	*	·OH			
**E**	-145.20845	-137.33142	-7.58395		△E	0.29308
**E_{ZPE}**	0.25561	0	0.22499		△E_{ZPE}	-0.03062
**S**	0.00015	0	0.00186		△S	0.00171
					△G	-0.2678965
Step (c)						
	*OH	·OH	*	H_2O_2		
E	-208.82925	-7.58395	-199.8305	-18.10111	△E	1.51841

**E_{ZPE}**	0.39361	0.22499	0	0.68914	△E_{ZPE}	-0.07054
**S**	0.00021	0.00186	0	0.00246	△S	-0.00039
					△G	1.5688285

Supplementary Table 2. The information on antibodies for all assays.

Antibodies used	
**Primary antibodies**	Phospho-Histone H2AX (Ser139) (D7T2V), Cell Signaling (Product # 80312), Dilution 1:200;<br>Cleaved Caspase-3 (Asp175) (5A1E), Cell Signaling (Product # 9664), Dilution 1:250;<br>8-hydroxy-2'-deoxyguanosine (8-OhdG), EpiQuik™ (Product # P-6003).
**Secondary antibodies**	Goat Anti-Rabbit IgG (H+L) Highly Cross-Adsorbed Secondary Antibody, Alexa Fluor 488, ThermoFisher (Catalog # A-11034), Dilution 1:1000;<br>Goat Anti-Mouse IgG (H+L) Highly Cross-Adsorbed Secondary Antibody, Alexa Fluor 647, ThermoFisher (Catalog # A-21236), Dilution 1:1000.
**Validation**	
**Primary antibodies**	Phospho-Histone H2A.X (Ser139) (D7T2V), Cell Signaling (Product # 80312), Dilution 1:200; IHC-Leica® Bond™ 1:200 - 1:800; Immunohistochemistry (Paraffin) 1:200 - 1:800; Immunofluorescence (Immunocytochemistry) 1:100 - 1:400; Flow Cytometry; Species Reactivity: Human, Mouse, Rat, Monkey.<br>Cleaved Caspase-3 (Asp175) (5A1E), Cell Signaling (Product # 9664), Dilution 1:250, validate for Western Blotting 1:1000; Immunoprecipitation 1:50; Immunohistochemistry (Paraffin) 1:2000; Immunofluorescence (Immunocytochemistry) 1:400 - 1:1600; Flow Cytometry; Species Reactivity: Human, Mouse, Rat, Monkey.<br>Alidation details of the 8-hydroxy-2'-deoxyguanosine (8-OhdG) according to validation statements are available on the manufacturers' websites: https://www.epigentek.com/catalog/index.php.
**Secondary antibodies**	Goat Anti-Rabbit IgG (H+L) Highly Cross-Adsorbed Secondary Antibody, Alexa Fluor 488, ThermoFisher (Catalog # A-11034), Dilution 1:1000; Immunohistochemistry (IHC), Immunohistochemistry (Paraffin) (IHC (P)), Immunohistochemistry (Frozen) (IHC (F)), Immunohistochemistry - Free Floating (IHC (Free)), Flow Cytometry (Flow), Immunocytochemistry (ICC/IF), Miscellaneous PubMed (Misc).<br>Goat Anti-Mouse IgG (H+L) Highly Cross-Adsorbed Secondary Antibody, Alexa Fluor 647, ThermoFisher (Catalog # A-21236), Dilution 1:1000. validate for Western Blot (WB), Immunohistochemistry (IHC), Immunohistochemistry (Paraffin) (IHC (P)), Flow Cytometry (Flow), Immunocytochemistry (ICC/IF), Miscellaneous PubMed (Misc)
